# Concertina Phenomenon in the Left Anterior Descending Artery: An Unexpected Circumstance in an Unexpected Vessel

**Published:** 2018-10

**Authors:** Mert İlker Hayıroğlu, Tufan Çınar, Ahmet Öz, Muhammed Keskin

**Affiliations:** *Haydarpaşa Sultan Abdülhamid Han Training and Research Hospital, Istanbul, Turkey.*

**Keywords:** *Coronary vessels*, *Coronary angiography*, *Coronary stenosis*

## Abstract

The concertina phenomenon is the occurrence of new and transient angiographic series of pseudolesions in a tortuous vessel induced mainly by a stiff guide wire. Here, we describe a 53-year-old man who experienced a concertina effect in the left anterior descending coronary artery (LAD) during an elective percutaneous coronary intervention. After the diagnosis of the concertina phenomenon in the LAD, a percutaneous coronary intervention was performed following the withdrawal of the soft guide wire to the mid level of the LAD. After the intervention, the patient remained in very good clinical status and was discharged on the third postprocedural day.

## Introduction

The concertina (or accordion) phenomenon (CP) is the occurrence of new and transient angiographic pseudolesions or stenoses after the placement of a stiff guide wire in a tortuous artery.^1^ Here, we present a case report of this phenomenon in the left anterior descending coronary artery (LAD) induced by a soft guide wire.

## Case Report

**Figure 1:**
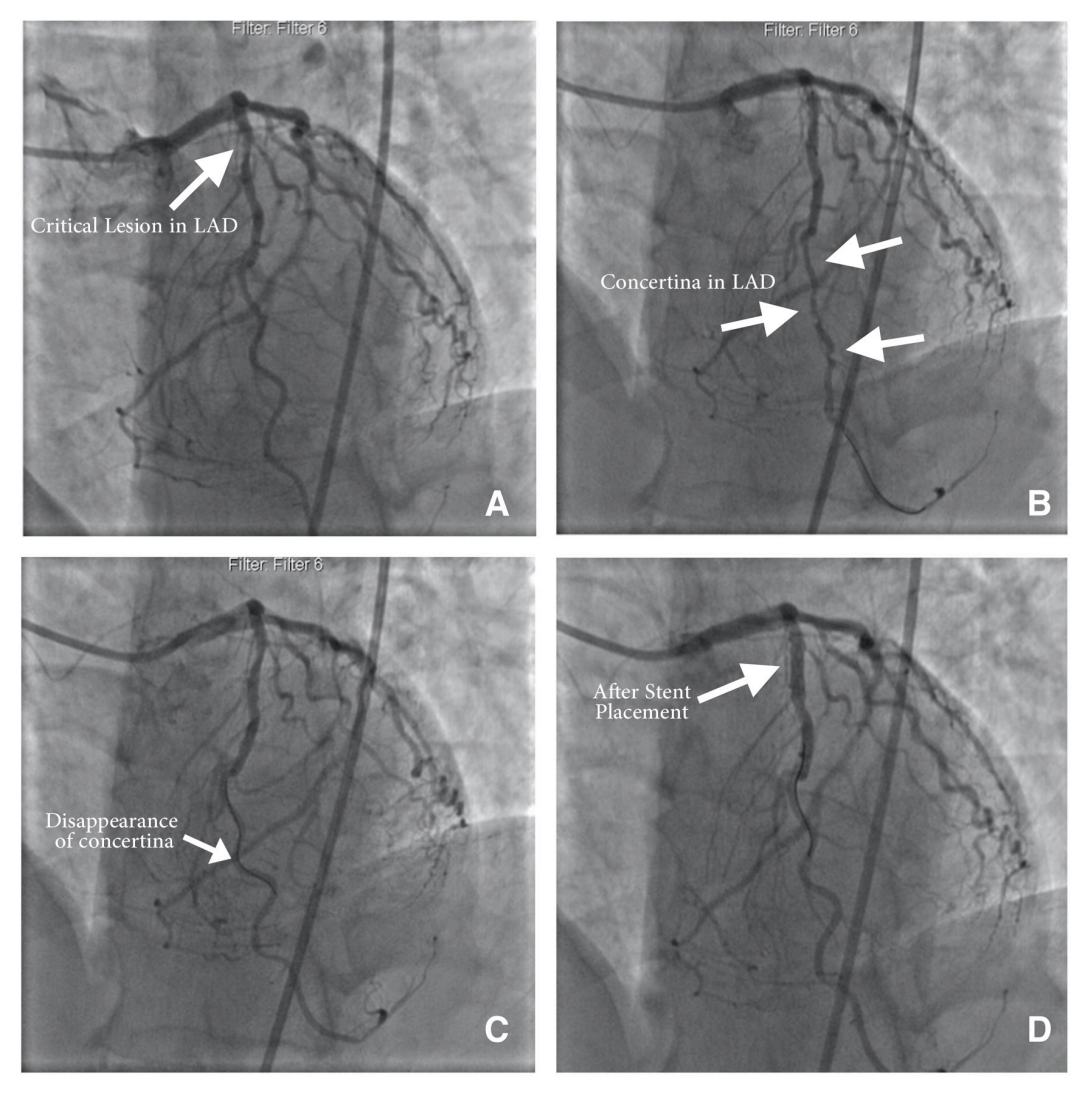
A) Critical lesion found in the proximal portion of the left anterior descending artery (LAD) in the left anterior oblique (LAO) image. B) Persistence of the concertina effect despite multiple doses of intracoronary nitrates while the floppy guide wire is still in place in the LAO image. C) After the placement of the guide wire in the mid portion of the LAD, note the disappearance of the mid and distal pseudolesions (concertina effect) in the LAO image. D) Final angiographic view of the LAD after the placement of the stent and the withdrawal of the guide wire in the LAO image.

A 53-year-old male patient presented to our cardiology department with a retrosternal chest pain of 2 months’ duration. On medical history, the patient was diagnosed with hypertension 2 years previously and was prescribed a valsartan–amlodipine combination. Electrocardiography revealed a normal sinus rhythm without ischemic findings. The blood pressure of the patient was 130/80 mm Hg. On physical examination, auscultation of the chest showed no murmurs or pathologic sounds and the other systems were normal. Transthoracic echocardiography demonstrated a normal left ventricular systolic function, mild mitral regurgitation, and grade 1 diastolic dysfunction. The exercise stress test yielded a Duke treadmill score of -12. Hence, coronary angiography was scheduled and performed via the femoral artery using a 6-F Judkins left diagnostic catheter. The results showed a critical stenosis in the proximal LAD (Figure 1A). Therefore, a decision was made to perform a percutaneous coronary intervention on the proximal portion of the LAD. Following the decision, a 6-F Judkins left guiding catheter was passed through the femoral artery to the ostium of the left main coronary artery. Before wiring, intracoronary nitroglycerine was used in order to exclude vasospasm. A choice floppy guide wire (Boston Scientific, Natick, MA, USA) was used, and its 3-cm radiopaque tip was placed in the distal part of the LAD. The wiring was followed by the occurrence of pseudolesions (the concertina effect) at the mid and distal segments of the LAD and the disappearance of the proximal LAD lesion. The disappearance of the true lesion was considered to be secondary to the CP (Figure 1B). Interestingly, the LAD did not have a high tortuous course. The pseudolesions were refractory to the intracoronary nitroglycerine injection. The choice floppy guide wire was withdrawn since the proximal critical lesion was lost in the angiographic images secondary to the accordion effect. The pseudolesions disappeared after the choice floppy guide wire was placed in the mid LAD (Figure 1C). A 3.0 × 16 mm PROMUS Element Stent (Boston Scientific, Natick, MA, USA) was deployed at 14 atm in order to prevent ischemic arrhythmias (Figure 1D). After the intervention, the patient remained in very good clinical status and was discharged on the third postprocedural day.

## Discussion

The CP is described as the occurrence of new lesions or stenoses after the placement of a stiff guide wire in a tortuous artery.^1^ The CP is usually seen as a result of the straightening of the tortuous segment of a coronary artery. The right coronary artery is the most commonly affected coronary artery as it is located in the epicardial fat tissue and courses rather freely in the atrioventricular groove.^2^ The traditional risk factors associated with coronary spasms such as cigarette smoking and hypertension are also related to this phenomenon.^3^ The 2 most important risk factors for the occurrence of this phenomenon are an increased tortuosity of the vessel and the use of stiff interventional guide wires. 

The CP effect usually has no major clinical sequelae and does not usually require any special interventions; however, it may cause hemodynamic compromise and ischemia. The differential diagnosis plays a major role in patient management during the CP. If the interventional cardiologist cannot recognize the CP, it may cause unnecessary further percutaneous coronary interventions to an otherwise normal coronary segment.^4^ In the literature, there are reported cases of coronary CP mimicking coronary dissection.^5^^, ^^6^ In contrast to the reported cases in the literature, our case had more interesting points insofar as not only was the CP observed in consequence of the use of a soft guide wire but also it occurred in an unexpected vessel (the LAD), even in the absence of a tortuous course. As was the case in our patient, the removal of a guide wire and the prompt stenting of an originally stenosed segment are vital to the prevention of ischemia or arrhythmia.

## Conclusion

Even though the concertina phenomenon is a rare entity, interventionists must be aware of this phenomenon and recognize it since it might lead to unnecessary procedures.

## Notes:

***This paper should be cited as: ***İlker Hayıroğlu M, Çınar T, Öz A, Keskin M. Concertina Phenomenon in the Left Anterior Descending Artery: Unexpected Circumstance in an Unexpected Vessel. J Teh Univ Heart Ctr 2018;13(4):183-185.
